# Tuméfaction du muscle pectoral révélant une tuberculose musculaire isolée

**DOI:** 10.11604/pamj.2017.27.44.12419

**Published:** 2017-05-18

**Authors:** Sohaib Hayoun, Hanane El Ouazzani, Bouchra Habibi, Salwa Belhabib, Hicham Souhi, Ismail Abderahmane Rhorfi, Ahmed Abid

**Affiliations:** 1 Service de Pneumologie, Hôpital Militaire et d’Instruction Mohammed V, Rabat, Maroc; 2 Service d’Anatomopathologie, Hôpital Militaire et d’Instruction Mohammed V, Rabat, Maroc

**Keywords:** Tuberculose, muscle, tuméfaction, Tuberculosis, muscle, swelling

## Abstract

La tuberculose des parties molles représente l’une des formes rares de la tuberculose extra pulmonaire, la localisation musculaire isolée est par-ailleurs exceptionnelle. Nous rapportant une observation médicale originale, une tuberculose musculaire isolée intéressant le muscle grand pectoral chez un jeune patient immunocompétent. Le diagnostic a reposé essentiellement sur l’histologie. L’évolution été favorable sous traitement anti bacillaire seul. Ce cas rare est présenté avec revue de littérature.

## Abstract (en)

Soft tissue tuberculosis is one of the rare forms of extrapulmonary tuberculosis. Furthermore, isolated muscular tuberculosis is exceptional. We here report the original case of a young immunocompetent patient with isolated muscular tuberculosis involving major pectoralis muscle. The diagnosis was mainly based on histology. The patient was successfully treated with anti bacterial therapy only. This rare case study has been combined with a literature review.

## Introduction

Le Maroc représente un des pays à forte endémie de tuberculose, la localisation extra pulmonaire a connu une nette augmentation cette dernière décennie. L'atteinte musculaire sélective reste une forme très rare de la tuberculose extra-pulmonaire même dans les pays à forte endémie de la maladie. L'atteinte du muscle grand pectoral fait partie des atteintes de la paroi thoracique qui représentent 1 à 5% des atteintes musculo-squelettiques [1]. Le tableau clinique est souvent trompeur devant l'absence des signes d'appel généraux ou pleuro-pulmonaires. L'imagerie n'est pas univoque. Le diagnostic est basé surtout sur la bactériologie et l'histologie. Le traitement fait appel aux anti-bacillaires seules ou le plus souvent associés à une chirurgie d'exérèse ou de drainage.

## Patient et observation

**Figure 1:**
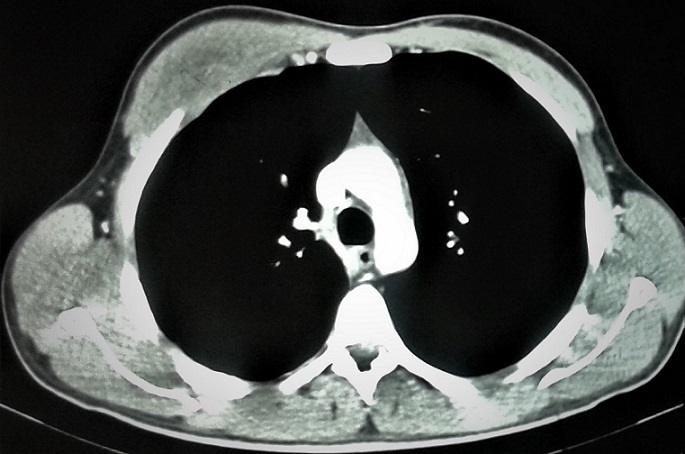
Coupe scannographique objectivant une tuméfaction du muscle grand pectoral droit

**Figure 2:**
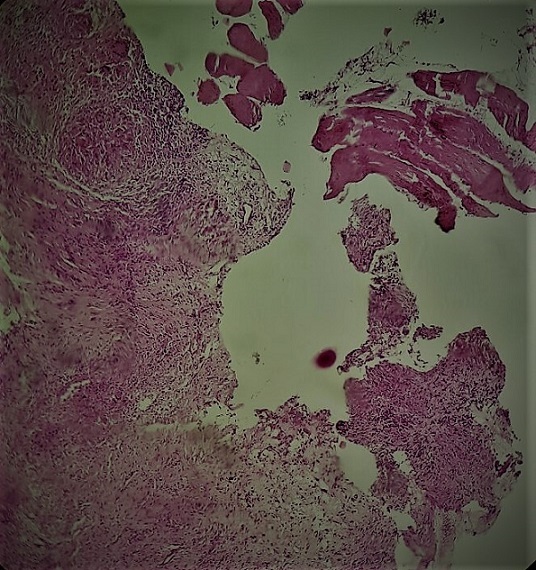
Coupe histologique montrant des granulomes épithélioïde giganto-cellulaire avec nécrose caséeuse (après fixation par le formol et inclusion en paraffine et coloration HE) Gx40

**Figure 3:**
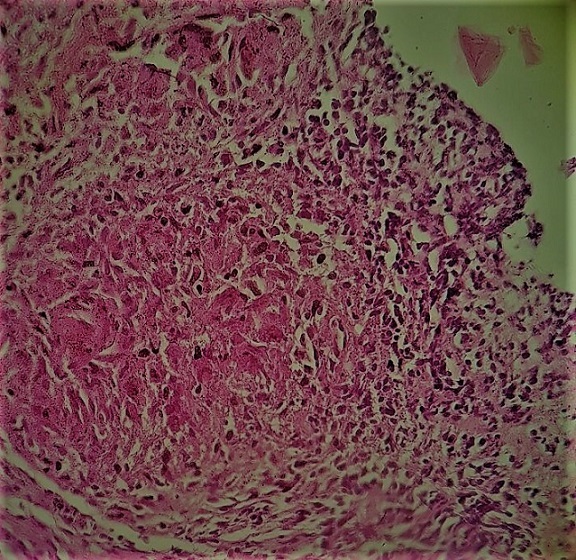
Coupe histologique montrant des granulomes épithélioïde giganto-cellulaire avec nécrose caséeuse (après fixation par le formol et inclusion en paraffine et coloration HE) Gx100

**Figure 4:**
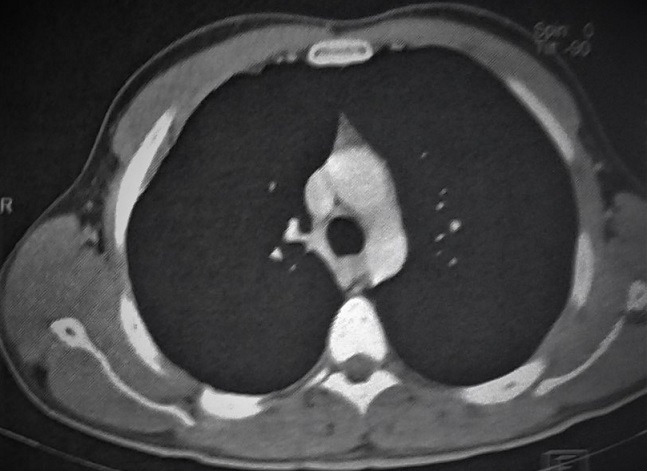
Coupe scannographique à la fin du traitement anti-bacillaire montrant la disparition quasi complète de la masse pectorale droite

Un patient de 29 ans, jamais traité pour tuberculose, sans notion de contage tuberculeux récent, et sans aucun antécédent de traumatisme thoracique ou antécédent pathologique connu, présente depuis quatre mois une tuméfaction de la partie antéro-supérieure droite de la paroi thoracique, augmentant progressivement de volume, évoluant dans un contexte d'apyrexie et de conservation de l'état général. L'examen clinique met en évidence une augmentation homogène du volume du muscle grand pectoral droit de consistance dure, indolore et sans signes inflammatoires en regard. La radiographie thoracique est sans particularités. L'échographie thoracique montre une masse tissulaire hétérogène faiblement vascularisée au doppler couleur de 2cm d'épaisseur siégeant sous le muscle grand pectoral droit sans épanchement pleural liquidien associé. La TDM thoracique montre un épaississement des parties molles en regard du muscle grand pectoral droit associé à un épaississement pleural sous jacent sans lésions parenchymateuses pulmonaires associées (Figure 1). Le bilan biologique était strictement normal notamment une VS à 20mm à la première heure, une CRP à 12mg/l, des globules blancs à 8700/ml avec des lymphocytes à 2000/ml, et la sérologie HIV était négative. Une ponction biopsie de cette masse était réalisée sous repérage échographique, l'analyse bactériologique des fragments obtenus à la recherche de bacille de koch s'est révélée négative, alors que l'examen anatomo-pathologique était en faveur d'une granulomatose spécifique avec nécrose caséeuse (Figure 2, Figure 3). Le patient était mis sous traitement anti-bacillaire pendant 6 mois selon le schéma 2RHZE/4RH avec une bonne évolution radio-clinique marquée par la régression quasi complète de la masse pectorale (Figure 4).

## Discussion

La tuberculose est une maladie granulomateuse qui peut toucher tous les organes, elle se manifeste sous différentes formes. L'atteinte musculaire isolée ou primaire est une forme très rare de la tuberculose extra-pulmonaire. Son incidence exacte n'est pas connue. Peu d'études ont abordées cette localisation exceptionnelle du mycobactérium tuberculosis, les connaissances actuelles sont essentiellement fondées sur l'analyse des cas sporadiques. Dans la série de Culotta A, seulement 4 cas de tuberculose musculaire ont été révélés sur 2224 autopsies réalisées chez des patients tuberculeux [2]. Une étude plus récente réalisée en Taiwan a mis en évidence 21 cas de tuberculose musculaire sur un total de 1153 patients tuberculeux avec culture positive, soit un taux d'environ 2% [3]. L'atteinte musculaire intéresse surtout les muscles de la cuisse, les bras et avant-bras et les mollets [4]. A notre connaissance, l'atteinte du muscle grand pectoral comme le cas de notre patient n'a jamais été rapportée dans la littérature. L'atteinte musculaire peut se présenter sous forme de masse kystique rénitente, indolore sans signes inflammatoires en regard réalisant un tableau d'abcès froid ou de masse tissulaire ferme parfois mobile [5]. La physiopathologie de cette localisation n'est pas claire. La concentration élevée en acide lactique, ainsi que la riche vascularisation sanguine et l'absence du tissu réticulo-endothélial et lymphatique dans le muscle, font de ce dernier un milieu défavorable à la croissance du mycobacterium tuberculosis [6], Ce qui explique que l'atteinte musculaire dans la tuberculose provient habituellement de l'extension d'un foyer osseux, articulaire ou tendineux, d'une inoculation directe ou d'une dissémination hématogène ou lymphatique [7]. La localisation musculaire isolée comme dans notre cas où aucune localisation primitive n'a été détectée et aucune notion d'inoculation iatrogène n'a été rapportée est extrêmement rare et elle serait secondaire à une fragilisation des muscles par un traumatisme ou une affection préexistante méconnue [8].

Cependant, la contamination aurait pu se faire à partir d'un foyer pulmonaire radiologiquement indécelable. Dans une région de forte endémie tuberculeuse, le diagnostic peut être évoqué devant un faisceau d'arguments cliniques avec un antécédent de tuberculose pulmonaire, des signes d'imprégnation tuberculeuse et une tuméfaction pariétale augmentant progressivement de volume. La radiographie peut montrer un aspect de masse kystique ou tissulaire renfermant une zone d'hypodensité centrale et un rehaussement périphérique en anneau. La TDM thoracique est l'examen de choix dans l'évaluation des lésions de la tuberculose de la paroi thoracique en montrant la nature et l'étendue de ces lésions, l'existence d'une lyse osseuse, d'adénopathie intra thoracique ou de lésions pleuro pulmonaires associées. L'imagerie par résonance magnétique (IRM) avec sa capacité multiplanaire et son excellent contraste pour les tissus mous reste le meilleur examen radiologique pour évaluer les masses des tissus mous, y compris les processus infectieux et inflammatoires [9]. Le diagnostic différentiel reste difficile vu la rareté de la tuberculose musculaire isolée. Cependant il est important d'éliminer une myosite ossifiante dans sa forme localisée survenant surtout chez le sujet jeune le plus souvent à la suite d'un traumatisme, et le sarcome des parties molles dont l'aspect peut être trompeur. Le diagnostic de certitude reste bactériologique en montrant la présence de bacille de Koch à l'examen direct et à la culture ou bien histologique en montrant un granulome spécifique avec nécrose caséeuse. Le prélèvement peut être obtenu par ponction-aspiration à l'aiguille ou par voie chirurgicale.

Dans notre cas le diagnostic histologique était obtenu par biopsie chirurgicale sous anesthésie locale. Le traitement optimal n'est pas bien codifié. La durée du traitement médical, la nécessité et les modalités d'un éventuel traitement chirurgical sont les principaux points de débat. Le schéma fait de 2 mois de rifampicine-isoniazide-pyrazinamide-Ethambutol suivi de 4 mois de rifampicine-isoniazide reste le standard pour les atteintes musculaires pures épargnant l'os. Dans les cas contraire ou lorsque les atteinte sont diffuses une prolongation du traitement peut s'avérer nécessaire [10]. Certains auteurs suggèrent qu'un traitement médical seul bien conduit peut mener à la guérison, Cependant, il s'agit de rares séries avec un nombre imité de patients, un suivi court et un taux élevé de récidives. La plupart des auteurs recommandent la combinaison d'un traitement médical et d'une cure chirurgicale. Notre cas a bien répondu au traitement médical exclusif délivré pendant 6 mois. L'évolution était spectaculaire avec régression presque complète de la masse et absence de récidive avec un recul de 3 ans.

## Conclusion

Bien que la tuberculose musculaire isolée soit une entité rare, elle doit être toujours prise en considération comme diagnostic différentiel devant toute tuméfaction musculaire isolée surtout dans un pays à forte endémie de tuberculose. Le diagnostic est surtout bactériologique et/ou anatomopathologiques. Le traitement fait appel à la chimiothérapie anti-bacillaire seule ou associée à la chirurgie. Le pronostic est souvent favorable lorsque la prise en charge est précoce.

## Conflits d’intérêts

Les auteurs ne déclarent aucun conflit d'intérêts.
